# 778. PrEParing Medical Students For Their Transition to Residency: The Benefit of Early Education on HIV Prevention

**DOI:** 10.1093/ofid/ofad500.839

**Published:** 2023-11-27

**Authors:** Jonathan E Pavia, Joseph P Hornak

**Affiliations:** University of Texas Medical branch, Leauge City, Texas; University of Texas Medical Branch at Galveston, Leauge City, Texas

## Abstract

**Background:**

Pre-exposure prophylaxis **(**PrEP) is a cornerstone of broad efforts to end the HIV epidemic, but uptake by primary care providers (PCP) has been demonstrated as suboptimal. Knowledge gaps have been linked to decreased PrEP uptake among PCPs. This report demonstrates the effectiveness of early career PrEP education aimed at the graduating 4th year medical student (MS4) as they transition to residency.

**Methods:**

Anonymous, online, optional, pre- and post-intervention surveys were administered to MS4s at our institution to assess awareness, knowledge, and perceived comfort around prescribing PrEP as future resident physicians. Students attended a comprehensive, interactive, case-based lecture (“intervention”) about HIV prevention, with particular focus around PrEP availability, identification of potential candidates, and patient monitoring. Results from the two surveys were measured and compared. Fisher's exact test was used for nominal statistics.

**Results:**

Thirty-one pre-intervention and 19 post-intervention surveys were completed. At baseline, a majority of respondents were aware of PrEP (94%), but a minority were comfortable prescribing PrEP (39%). Prior knowledge regarding PrEP (67%) and concerns for cost (16%) were the two highest reported factors contributing to perceived comfort with prescribing PrEP. Post-intervention, MS4s reported significantly increased comfort prescribing PrEP (100% vs. 39%, *P* = < 0.0001) and no significant decrease in attributing knowledge as a barrier to providing PrEP (52% vs. 67%, *P* = 0.37). Participants reported very high satisfaction in the material presented during the lecture (100%).

**Conclusion:**

These results indicate the benefit of early career education on the topic of HIV PrEP aimed at senior medical students. If efforts to significantly reduce HIV transmission are to be successful, PrEP use must be greatly expanded across medical disciplines including primary care, pediatrics, infectious diseases, women’s health, and others. Bolstering PrEP education within medical school curricula may assist with accomplishing these goals. Further research should explore other modalities for delivering HIV prevention education to medical students.

**Disclosures:**

**All Authors**: No reported disclosures

